# Transcatheter intervention of modified Blalock–Taussig shunts in patients with hypoplastic left heart syndrome undergoing stage 1 palliation

**DOI:** 10.3389/fcvm.2024.1502801

**Published:** 2024-11-20

**Authors:** Nathalie Mini, Martin B. E. Schneider, Marian Mikus

**Affiliations:** ^1^ Cardiac Catheterization Laboratories, Department of Pediatric Cardiology, German Pediatric Heart Center, University Hospital Bonn, Bonn, Germany; ^2^ Department of Anesthesiology and Intensive Care Medicine, University Hospital Bonn, Bonn, Germany

**Keywords:** Norwood I, hypoplastic left heart syndrome, shunt stenting, shunt stenosis, mBT shunt, ECMO, shunt thrombosis

## Abstract

**Background:**

While several studies have explored the outcomes of transcatheter interventions for modified Blalock–Taussig shunts (MBTSs) in a broad range of congenital heart diseases, none have specifically examined the interventions in patients with hypoplastic left heart syndrome (HLHS) who underwent Norwood palliation (NP).

**Methods:**

This retrospective study was conducted between 2020 and 2024, when 24 urgent interventions were performed on 17 patients at our center. We recorded several key outcomes, including early and late intervention-related complications, the need for reintervention, the interval between the NP and the first intervention, shunt patency following the intervention, associated morbidities, and thrombosis-related sudden events. Additionally, during follow-up, we documented the outcomes for patients who underwent the Glenn procedure and those who were palliated, including late death.

**Results:**

The median age and weight at the time of intervention were 88 days (range: 15–300 days) and 5 kg (range: 2.6–7.6 kg), respectively. The median interval between the Norwood procedure and the transcatheter intervention was 61 days (7–160 days), with median shunt patency lasting 62 days (1–150 days). Notably, there were no intervention-related complications or deaths. In-stent thrombosis, a late complication, occurred in four patients; two of these had impaired anticoagulation, including extracorporeal membrane oxygenation (ECMO)-related causes, while the other two, who were on aspirin, had multiple stents within the shunt, one of whom experienced sudden death. Six patients required seven reinterventions: four due to shunt obstruction or restenosis and two to delay surgery or provide palliation for patients unfit for surgery, aiding in pulmonary development.

**Conclusion:**

The transcatheter intervention of the MBTSs in patients with HLHS undergoing NP is considered both safe and potentially life-saving in emergent situations. This approach may facilitate improved pulmonary development, postpone the need for subsequent surgeries, and provide medium-term palliative care for critically ill patients. However, managing late complications such as stent thrombosis remains a significant challenge. Our findings indicate that risk factors for shunt thrombosis include using ECMO therapy, underlying coagulation disorders, impaired lymphatic drainage, requiring multiple stents within the shunt, and a prior history of thrombosis before intervention. Dual antiplatelet therapy is recommended to alleviate the risk of thrombotic events in this population.

## Introduction

Hypoplastic left heart syndrome (HLHS) is a rare congenital heart defect that severely disrupts normal blood flow through the heart and carries a high risk of mortality if untreated, with survival rates of only 30% in the first week and 9% by 30 days (1). Norwood palliation (NP) is typically the first stage of palliation for HLHS patients.

Both early and late shunt obstructions significantly contribute to the morbidity and mortality of patients who undergo NP with a modified Blalock–Taussig shunt (MBTS) (2–4). Shunt stenosis or obstruction can arise from various factors, including intraluminal thrombosis, neointimal hyperplasia, suture line stricture, and vascular distortion due to surgery or ductal tissue constriction (5–7). These issues can lead to a gradual decline in oxygen saturation and exercise tolerance or result in rapid clinical deterioration characterized by acute hemodynamic instability and severe hypoxemia, which can become life-threatening (8).

The severity of shunt stenosis can vary widely, from mild to critical, often necessitating urgent intervention. The primary goal of transcatheter interventions for stenosed or obstructed shunts is to restore and maintain shunt patency, ensuring adequate pulmonary blood flow and minimizing the risk of cyanosis and other complications. However, these interventions are not without risk; complications such as cardiac tamponade, catheter-induced arrhythmias, atrioventricular block, thromboembolic stroke, and vascular access injuries have been reported (9).

Given the high rates of morbidity and mortality associated with HLHS, this study focused on shunt interventions within this patient cohort. We will review our results and outcomes, emphasizing the impact of associated morbidities on procedural success.

## Patients and methods

We conducted a retrospective analysis of 17 patients with HLHS who underwent NP and received 24 transcatheter interventions on MBTSs between January 2020 and May 2024. We documented several important outcomes, such as complications related to early and late interventions, the need for reintervention, the time elapsed between NP and the first intervention, post-procedure shunt patency, associated morbidities, and thrombosis-related sudden events. Furthermore, during follow-up, we tracked outcomes for patients who underwent the Glenn procedure and those who received palliative care, including cases of late mortality.

Table 1 summarizes patients' demographics, including age and weight at the time of intervention and the indications for emergent procedures. Table 2 summarizes the morbidities associated with the intervention. Echocardiographic assessments were performed 24 and 48 h post-intervention to monitor for any pericardial or pleural effusions. A previous study published a detailed description of the intervention techniques and the patients' management after the procedure in our center (10). In small children weighing under 4 kg, the stent size was equal the shunt size to prevent secondary pulmonary overcirculation. In patients weighing >4 kg who have contraindications for surgery in the usual time (age of 5–6 months) and will need the shunt longer than others, the stent size was 1 mm more than the shunt size.

**Table 1:** Patients’ descriptions and the indications and outcomes of the shunt interventions.

N	NW/I interval (days)	Shunt Size (mm)	Indication	Stenting/dilation	Size of stent/balloon (mm)	Reinterventions	Outcome	Late death?
1	160	4	SS	Stenting	4		TKR + aortic arch reconstruction	Death 7 days postoperatively
2	55	3.5	Desaturation by SS and severe PA hypoplasia	Stenting	3.5	1. A new stent (4 mm) 120 days after the first one 2. Recanalization of the deformed stent after CPR 55 days after the second one	Hypoxic encephalopathy/Palliative	Death in a rehabilitation center
3	123	3.5	SS + severe PA hypoplasia	Stenting	4		Glenn	
4	74	4	SS + PAPVR	Stenting	4.5	A new shunt stent (5 mm) will delay the operation 70 days after the first stent.	No clear plan by associated PAPVR/operation was delayed/palliation	Sudden death 110 days after the first stent and 40 days after the second one
5	69	3.5	SS + PAVM	Stenting	3.5		Glenn	
6	81	3.5	SS	Stenting	4		Glenn	
7	8	3.5	Shunt obstruction postoperatively by severe ECMO-related thrombosis	Recanalization and Stenting on ECMO	3.5		Shunt revision by disseminated thrombosis on ECMO and secondary shunt-stent obstruction	Death 7 days after the shunt revision on ECMO
8	61	3.5	SS	Stenting	3.5		Glenn	
9	270	4	SS + hyp. PAs and PAVM	Stenting	4.5		Palliative	
10	7	3.5	SS + PAS	Stenting	4		Glenn takedown/Re-Glenn + TKR	
11	100	3.5	SS + PA hypoplasia	Stenting	4		3. Operation: TKR + PAR 4. Operation: Glenn	
12	61	3.5	SS	Stenting	3.5		Glenn	
13	62	4	S. obstruction postoperatively	Stenting	3.5	Recanalization of the obstructed shunt-stent	Palliative by thrombotic event-related neurologic problem	Death 60 days after the first intervention and 20 days after the second one
14	20	3.5	Desaturation by shunt clip with PA hypoplasia	Dilation	3.5	Shunt clip dilation	Palliative	
15	81	3.5	Desaturation with severe PA hypoplasia	Stenting	3.5		Improvement of PA development after stenting, the patient is waiting for Glenn	
16	15	3.5	Severe SS postoperatively with sharp angulation of the shunt	Dilation	3.5	Stenting to delay the operation by CoA/impaired RV 100 days after the first intervention	Glenn + shunt/aortic arch. Reconstruction + long ECMO-Therapy	
17	13	3.5	SS postoperatively	Dilation	3.5	Shunt clip redilation (4 mm) by desaturation 32 days after the first one	Glenn	

NW/I interval between Norwood palliation and the intervention time. CoA, aortic coarctation; CPR, cardiac pulmonary resuscitation; MBTS, modified Blalock–Taussig shunt; PA, pulmonary; PAR, pulmonary reconstruction; PAS, pulmonary stenosis; PAVM, pulmonary arteriovenous malformation; SS, shunt stenosis; TKR, tricuspid reconstruction; Hyp.; PA, pulmonary hypoplasia.

**Table 2:** Illustrates the morbidities associated with shunt interventions, along with the antiplatelet therapy administered following the procedure.

N	Aortic arch stenosis torsion?	TR?	Need for reoperation?	PA hypoplasia? PAS? PAVM? PAPVD?	Coagulation pathway?	Antiplatelet	Sudden life-threatening event?
1	Torsion	Yes	Yes	No	No	Heparin	
2	CoA	No	No	Bilateral hypoplasia	Yes, on ECMO	Aspirin	Yes
3	No	No	No	Bilateral hypoplasia	No	Aspirin	
4	No	No	No	PAPVR	No	Aspirin	Yes
5	No	No	No	Shunt related surgical RPA stenosis (torsion)/right PAVM	No	Aspirin	
6	No	No	No	No	No	Aspirin	
7	No	No	Yes	No	Yes, on ECMO	Enoxapam	Yes
8	No	No	No	No	No	Aspirin	
9	No	No	Yes	Shunt related RPA stenosis (torsion)/right PAVM	No	Aspirin	
10	No	Yes	Yes	No	No	Aspirin	
11	No	No	No	Bilateral hypoplasia	No	Aspirin	
12	No	No	No	No	Yes	Aspirin	
13	No	No	No	No	Factor VII	Heparin	Yes
14	No	No	No	Bilateral stenosis	No	Aspirin	
15	No	No	No	Severe hypoplasia (bilateral)	No	Aspirin + Clopidogrel	
16	Arch torsion + CoA	No	Yes	No	No	Xarelto	
17	No	No	No	No	No		

CoA, aortic coarctation; PA, pulmonary; PAPVC, partial abnormal pulmonary venous return; PAS, pulmonary stenosis; PAVM, pulmonary arteriovenous malformation; TR, tricuspid regurgitation.

The Glenn operation is conducted at our center for patients weighing ≥ 5 kg who satisfy the following criteria: well-developed central and lower lobes of the pulmonary arteries, as indicated by a total lower lobe index (TLLI) > 95 mm^2^/m^2^; normal pulmonary pressure; normal wedge pressure; absence of significant pulmonary vein stenosis; normal diastolic pressure of the right ventricle with satisfactory function; and no evidence of coagulopathy.

### Statistical analysis

All statistical analyses were performed using SPSS version 22. Continuous variables are reported as median ± IQR, and categorical variables as count (percentage).

### Ethical statement

According to the local ethical committee's decision (with running number 2024-406-BO), informed consent and patient agreement were waived due to the study's retrospective design.

## Results

In our study, we performed 24 transcatheter interventions across 17 patients with HLHS, four of which involved shunt clip dilations in three patients. The median age and weight at the time of intervention were 88 days (range 15–300 days) and 5 kg (range 2.6–7.6 kg), respectively. The median interval between the Norwood procedure and the transcatheter intervention was 61 days (range 7–160 days), while the median duration of shunt patency was 62 days (range 1–150 days). Notably, there were no intervention-related complications or deaths, and follow-up echocardiography did not reveal any pericardial or pleural effusions.

### Emergent interventions for obstructed shunts

Among the 17 patients, two (12%) required emergent recanalization of obstructed shunts with stenting due to extracorporeal membrane oxygenation (ECMO)-related thrombotic events (Patient 7) and Factor VII deficiency (Patient 13), who had experienced multiple thrombotic incidents, including cerebral and systemic vein thrombosis. The shunt patency lasted 1 day for Patient 7 and 20 days for Patient 13. During follow-up, both patients experienced stent occlusions due to new thrombotic events, necessitating additional interventions: shunt and ECMO revision for Patient 7 and new recanalization for Patient 13.

### Emergent postoperative interventions

Approximately 29% (five patients) required urgent interventions due to shunt-related stenosis following surgery. Three patients (18%) underwent adequate shunt clip dilation, significantly improving oxygen saturation. Two of these patients (Patients 14 and 17) required end-size redilation, while the third patient (Patient 16) needed stenting to delay further surgery due to right ventricular dysfunction. Additionally, two other patients required stenting, including the previously mentioned one on ECMO.

### Interventions in patients with associated morbidities

Nine patients were deemed unfit for surgery due to severe pulmonary artery (PA) hypoplasia and stenosis, including one with tricuspid regurgitation (TR) and one with atypical partial anomalous pulmonary venous return (PAPVR). The goal of shunt stenting in these patients was to restore shunt patency, improve saturation, promote pulmonary development, and delay the Glenn surgery until pulmonary conditions were stable. Among five patients with PA hypoplasia, shunt stenting led to improved pulmonary parameters in four, of whom two underwent Glenn operations and one awaited surgery. The fifth patient was referred to palliative care.

In patients with PAVM, shunt interventions improved oxygen saturation but did not address the PAVM itself. One patient was referred to palliative care due to severe bilateral PAVMs and PA underdevelopment, while another successfully underwent a Glenn operation.

Patients with severe aortic arch torsion/stenosis had poorer outcomes. One underwent tricuspid and aortic reconstruction but died postoperatively. The other received aortic arch reconstruction and the Glenn procedure but later required additional shunt placement and extended ECMO therapy, ultimately resulting in palliative care.

### Reinterventions

Six patients (35%) required seven reinterventions due to acute shunt stent occlusion (in two patients, 12%), residual stenosis of the clipped shunt (in two patients, 12%), and dilation procedures for subsequent operations (in three patients, including Patient 3, who required two reinterventions).

### Thrombotic events and mortality

Thrombotic events occurred in four patients (24%), with two experiencing complications related to coagulation disorders from excessive ECMO-related transfusions (Patient 7) and Factor VII deficiency (Patient 13). The shunt stent patency was 1 day for Patient 7 and 40 days for Patient 13. Both were intensively anticoagulated with heparin.

The other two patients, Patients 2 and 4, received two stents in separate interventions. Patient 2 experienced sudden cardiac decompensation requiring ECMO, while Patient 4 suffered sudden death. Patient 2 was hospitalized at the time of the event, while Patient 4 was at home. Both patients were on aspirin. The first shunt stent patency lasted 120 days for Patient 2 and 100 days for Patient 4, while the second stent patency was 55 and 40 days, respectively.

### Follow-up

Nine patients underwent Glenn operations, including Patient 16, who required extended postoperative ECMO therapy. Five patients were referred to palliative care due to severe PA hypoplasia (three patients), a significant thrombotic event from Factor V Leiden (one patient), and atypical PAPVR (one patient). Of these, three died during follow-up. Two patients who underwent tricuspid valve replacement and shunt revision died 40 and 7 days postoperatively. At the time of writing, one patient with PA hypoplasia was still awaiting further surgery based on pulmonary artery development.

## Discussion

Despite improvements in operative mortality rates, complications arising from shunt failure continue to pose significant risks to patient morbidity and mortality. The estimated incidence of shunt failure for Blalock–Taussig shunts is approximately 9.3%, with early failures representing about 20% of these cases.

Numerous studies have explored the outcomes of transcatheter interventions for MBTSs, encompassing a broad spectrum of congenital heart diseases, including patients with HLHS. However (6, 9, 11), none have specifically focused on this particular cohort.

In our current report, we reviewed the results of transcatheter interventions for stenosed and obstructed MBTSs in this critical population, examining the associated morbidity and mortality that could influence outcomes.

Ensuring the MBTS's patency is crucial for the survival of patients with HLHS undergoing NP. Shunt failure can occur at various stages, driven by different mechanisms of obstruction. Acute obstructions are often associated with thrombus formation, while late obstructions typically arise from neointimal proliferation or calcification. In our experience, the median time to shunt failure or shunt intervention was about 2 months, although interventions were necessary at all points after shunt placement, including in critically ill patients supported by ECMO. This aligns with findings from a 2015 study that involved all patients who received MBTSs (*n* = 25), including those with HLHS (*n* = 10) (6).

In our cohort, the indications for shunt intervention in patients with HLHS undergoing NP were twofold: to restore the shunt lumen in cases of stenosis or obstruction or to postpone subsequent surgery in critically ill patients or those with underdeveloped pulmonary arteries. Although previous studies have reported some complications, all interventions performed in this critical cohort at our center over the past 4 years were free from procedure-related complications or deaths. Our study's median shunt patency after the intervention was approximately 2 months (64 days), including patients who underwent Glenn procedures at the optimal time and those who died due to non-stent-related causes. Shunt dilation and stenting were adequate in 76% of the patients in our cohort, providing palliative care or facilitating their progression to subsequent surgeries, such as the Glenn procedure or other necessary interventions like tricuspid valve reconstruction and aortic reconstruction. The first successful balloon angioplasty for shunt stenosis was documented in 1989 (12). This significant milestone led to further retrospective studies (10, 13, 14), which reported as high as 91% success rates. These findings align with our results, where the dilation of the shunt clip effectively improved oxygen saturation and served to bridge patients until the optimal time for subsequent surgery. Additionally, the dilation of the shunt clip in one patient was performed gradually to adjust the shunt size according to their weight. Approximately 24% of the patients experienced sudden thrombotic events, including instances of sudden death. In our cohort, the risk factors associated with thrombosis-related stent failure included a history of shunt occlusion, ECMO therapy necessitating transfusions, impaired coagulation pathways, and the presence of multiple stents within the shunt. Despite intravenous heparinization, preventing the recurrence of in-stent thrombosis in high-risk patients—especially those with impaired coagulation, including ECMO-associated thrombosis—remained challenging. In our cohort, aspirin alone was ineffective in preventing shunt thrombosis in patients with multiple stents, highlighting the need for dual antiplatelet therapy as a more suitable option for this population.

In a prior study conducted at our center (10), we investigated the outcomes of transcatheter MBTS interventions in patients with pulmonary atresia and those with HLHS. Our findings indicated that patients receiving MBTS solely to achieve adequate pulmonary perfusion—specifically those with tetralogy of Fallot, pulmonary atresia, and tricuspid atresia—exhibited better outcomes compared to those undergoing more complex procedures, such as the NP combined with MBTS. Among the patients who underwent MBTS alone in our study, 92% subsequently required additional surgical interventions: 46% received Glenn procedures, 38% underwent biventricular repair, and 8% required a Fontan procedure. Additionally, 8% of patients needed a Sano shunt. We observed significantly higher rates of shunt-related pulmonary torsion, surgery-related shunt stenosis, ECMO-related shunt thrombosis, and sudden death in the NP cohort compared to patients with pulmonary atresia who underwent a simple MBTS. These factors adversely affected the outcomes of MBTS interventions in the Norwood group compared to the other cohort.

The current results indicate that lower associated morbidities were correlated with better outcomes for patients who underwent shunt intervention. Morbidities such as tricuspid regurgitation, surgery-related aortic arch issues, pulmonary distortion, the need for additional operations, severe pulmonary atresia, and coagulation pathway disorders adversely affected the outcomes of patients in our cohort.

## Conclusion

The transcatheter intervention of MBTSs in neonatal patients with HLHS is recognized as both safe and potentially life-saving in emergencies. This method can enhance pulmonary development, delay the need for subsequent surgeries, and provide medium-term palliative care for critically ill patients. However, managing late complications, such as stent thrombosis, poses a significant challenge. Our findings suggest that the risk factors for shunt thrombosis include ECMO therapy, underlying coagulation disorders, requiring multiple stents within the shunt, and a history of shunt thrombosis before intervention. We recommend implementing dual antiplatelet therapy and performing comprehensive echocardiographic evaluations to detect shunt stenosis at an earlier stage, especially in high-risk patients. Additionally, it is essential to prevent dehydration and avoid the use of diuretics to reduce the risk of thrombotic events in this population.

## Data Availability

The original contributions presented in the study are included in the article/Supplementary Material, further inquiries can be directed to the corresponding author.
